# Concomitant Overactive Bladder Treatment and Adherence to Pelvic Floor Physical Therapy

**DOI:** 10.1007/s00192-025-06209-8

**Published:** 2025-07-22

**Authors:** Sarah Ashmore, Abigail Steinbeck, Nicholas Scioscia, Ashlee Weaver, Jessica C. Sassani

**Affiliations:** 1 Section of Urogynecology and Reconstructive Pelvic Surgery, University of Chicago, 1217 N California Ave, Apt 1W, Chicago, IL 60622 USA; 2 Department of Obstetrics and Gynecology, West Penn Hospital, Pittsburgh, PA USA; 3 Lake Erie College of Medicine, Erie, PA USA; 4 Department of Gynecology and Obstetrics, Johns Hopkins School of Medicine, Baltimore, MD USA; 5 Division of Urogynecology, Allegheny Health Network, Pittsburgh, PA USA; 6 Drexel University College of Medicine, Philadelphia, PA USA

**Keywords:** Concomitant medication, Overactive bladder, Pelvic floor physical therapy, Pelvic floor physical therapy adherence

## Abstract

**Introduction and Hypothesis:**

There is limited literature regarding concomitant initiation of pelvic floor physical therapy (PFPT) and medications for overactive bladder treatment. PFPT improves patient symptoms, although adherence tends to be low. This retrospective cohort study assessed PFPT adherence of female patients with overactive bladder at a tertiary care center who were referred to PFPT. We hypothesized that concomitant PFPT and medication would correlate with decreased PFPT adherence among patients with overactive bladder.

**Methods:**

Adherence to PFPT (defined as ≥ 50% attendance of the recommended sessions) was compared in patients with (PT + Med group) and in those without (PT group) concomitant medication prescription.

**Results:**

We evaluated 346 patients, with 196 in the PT group and 150 in the PT + Med group. The PT + Med group had a higher body mass index (30.0 kg/m^2^ vs 27.5 kg/m^2^, *p* < 0.001), a higher rate of diabetes (20.7% vs 11.7%, *p* = 0.02), and higher urogenital distress inventory scores at baseline (*p* < 0.001). The PT group completed more PT sessions (*p* < 0.001) and was more likely to be adherent (30.6% vs 15.3%, *p* < 0.001). The PT + Med group was more likely to progress to minimally invasive therapy (10.0% vs 4.1%, *p* = 0.03). On multivariable logistic regression model, PFPT adherence remained significantly lower for the PT + Med group when controlling for comorbidities (adjusted OR 0.38, *p* = 0.001).

**Conclusions:**

The addition of medication at the time of PFPT referral was associated with decreased PFPT adherence in overactive bladder patients.

## Introduction

Overactive bladder (OAB) is a syndrome characterized by urinary urgency, usually accompanied by frequency and nocturia with or without urinary incontinence [1]. OAB is estimated to affect 9% to 43% of women [1, 2] and can have detrimental consequences, including interference with daily activities, sleep disturbances, depression, and decreased quality of life [3–5]. Patients with OAB also have increased health care costs, with direct health care costs in the USA totaling more than $24 billion, annually [2, 6]. Current treatment guidelines for women with OAB include behavioral therapy and pharmacotherapy [1]. Behavioral therapy focuses on bladder training, toileting habits, dietary changes, pelvic floor physical therapy (PFPT), and biofeedback. Pharmacotherapy options include either anticholinergic medications or beta-3 agonists. The treatment guidelines previously supported a step-wise approach to treatment but are now encouraging shared decision making, which allows for concomitant initiation of behavioral and medication therapies for patients with OAB [1]. Here, we assessed the relationship between simultaneous treatment with OAB medication and PFPT and patients’ adherence to their PFPT regimen.

Pelvic floor physical therapy targets pelvic floor muscles to strengthen and improve coordination to suppress the micturition feedback mechanism [7]. PFPT alone has been shown to improve OAB symptoms; however, there is significant variability in outcomes experienced [8–10]. The effect of PFPT on patient outcomes depends on the length of training, improvement in pelvic floor muscle function, adherence to the exercise regimen, and side effects [10]. Previous studies have found that adherence to PFPT tends to be low, with fewer than 50% of women completing their prescribed PFPT treatment regimen [11–14]. There is limited literature regarding initiating both behavioral and medication therapy simultaneously, but studies suggest that it might improve outcomes [15, 16]. Mattiasson et al. found that OAB patients treated with a combination of anticholinergic medication and simplified bladder training had reduced voiding frequency and treatment satisfaction compared with patients treated with medication alone [16]. However, no previous study has evaluated the effect of concomitant OAB medication and PFPT on PFPT adherence.

In this study, we compared PFPT adherence in patients who were referred for PFPT alone and patients who were referred for PFPT with concomitant OAB medication. PFPT can take weeks to improve OAB symptoms [7], but we hypothesize that with the addition of OAB medication, patients might experience more rapid improvement. This rapid improvement may result in decreased patient perception of PFPT necessity and therefore, decreased PFPT adherence. We hypothesized that concomitant initiation of PFPT and medication might be associated with a decrease in PFPT adherence among patients with OAB.

## Materials and Methods

We performed a retrospective cohort study of female OAB patients with a PFPT referral who were seen by the Division of Urogynecology at a single tertiary care center between 1 January 2017 through 31 January 2024. Approval by the Institutional Review Board was obtained prior to case identification (IRB protocol approval 2020-380). We included female patients who were ≥ 18 years old with a PFPT referral for urgency urinary incontinence, urinary urgency, urinary frequency, OAB, or urge-predominant mixed urinary incontinence. ICD-10 codes were used to identify women with eligible diagnoses (N39.41, R35.0, R39.15, N32.81). We excluded pregnant patients and patients with a concomitant pelvic pain diagnosis, stress-predominant mixed urinary incontinence, or potential alternative etiology for OAB symptoms such as acute urinary tract infection. We also excluded patients who had undergone prior PFPT or minimally invasive OAB therapy, such as intravesical onabotulinumtoxinA, percutaneous tibial nerve stimulation, or sacral neuromodulation.

We reviewed patient electronic health records and collected baseline demographic, clinical, and outcome characteristics. We noted concomitant prescription or prior use of any OAB medication, including anticholinergic medications (oxybutynin, trospium, solifenacin, tolterodine, fesoterodine, and darifenacin) and beta-3 agonists (mirabegron or vibegron). Current or newly prescribed vaginal estrogen prescriptions were also documented, and all formulations were included. All patients underwent standard evaluation per established guidelines during their initial appointment [1]. Urogenital Distress Inventory (UDI) scores from the initial appointment were included as a proxy for baseline symptom bother. We calculated the need for 276 patients total, or 138 per group, to detect a significant change in adherence from 35% in the PFPT group to 20% in the PFPT with medication group using alpha 0.05 and power 80%.

Patients were separated into two groups: patients with only a PFPT referral (PT group) and patients with a PFPT referral and a prescription for an OAB medication (PT + Med group). We then compared adherence to PFPT in the two groups. Adherence was defined as $\ge$ 50% attendance of the recommended sessions. The recommended number of PFPT sessions for each patient was determined by the PT provider and abstracted from the initial PFPT session notes. Secondary outcomes included initiation of PFPT, completion of PFPT, and factors associated with PFPT adherence. Initiation of PFPT was defined as attending one or more PFPT sessions. Completion of PFPT was defined as attending all the recommended sessions or a final PFPT note from the provider stating that PFPT was no longer needed.

Statistical analyses were performed using Stata version 17 (StataCorp, College Station, TX, USA) with an alpha level of 0.05. Continuous variables were compared using a Student’s *t* test or Mann–Whitney *U* test, depending on data distribution. Categorical variables were analyzed using Chi-squared or Fisher’s exact tests where appropriate. Multivariable logistic regression was used to assess potential confounders. Backward selection of variables with *p* < 0.10 was used to identify variables for inclusion in the model. Final variables included body mass index (BMI), estrogen use, PT adherence, UDI score, and diabetes status.

## Results

A total of 346 patients received a PFPT referral and met the inclusion criteria. There were 196 patients in the PT group and 150 in the PT + Med group. Of those in the PT + Med group, 139 were newly prescribed mirabegron or vibegron whereas the other 11 were newly prescribed an anticholinergic OAB medication. Patients in the PT + Med group had a higher BMI (PT 27.5 kg/m^2^ vs PT + Med 30.0 kg/m^2^, *p* < 0.001), had higher rates of diabetes (PT 11.7% vs PT + Med 20.7%, *p* = 0.02), were more likely to be newly prescribed vaginal estrogen cream (PT 39.8% vs PT + Med 50.7%, *p* = 0.04), and reported higher UDI scores at baseline (PT 40 vs PT + Med 50, *p* < 0.001; Table 1). The median follow-up time was similar in the two groups at about 10 months (IQR 5–18.5, *p* = 0.30). Other demographic and clinical characteristics, including age, race, pelvic organ prolapse stage, and Charleson comorbidity index were also similar in the two groups (Table 1).

**Table 1:** Demographic and clinical characteristics in patients receiving physical therapy (PT) or physical therapy plus medication (PT + Med) for overactive bladder (OAB)

	PT group, *n* = 196	PT + Med Group, *n* = 150	*p* value
Age, years, median (IQR)	63.0 (53–70)	65.0 (55–71)	0.11
Race, *n* (%)			0.27
White	184 (93.9)	134 (89.3)	
Black	10 (5.1)	12 (8.0)	
Other or Unknown	2 (1.0)	2 (2.7)	
Urinary Distress Inventory score, median (IQR)	40 (25–50)	50 (33–60.5)	< 0.001
BMI, kg/m^2^, median (IQR)	27.5 (23.8–32.0)	30.0 (26.2–35.2)	< 0.001
Comorbidities			
Postmenopausal, *n* (%)	159 (82.0)	129 (86.0)	0.31
Current smoker, *n* (%)	12 (6.1)	5 (3.3)	0.32
Charleson comorbidity index, median (IQR)	3 (1–4)	3 (2–4)	0.07
Diabetes, *n* (%)	23 (11.7)	31 (20.7)	0.02
Treatment history, *n* (%)			
Prior hysterectomy	62 (31.6)	57 (38.0)	0.22
Pelvic organ prolapse, stage 3 or 4	14 (7.1)	5 (3.3)	0.16
Vaginal estrogen, initiated at time of PT referral	78 (39.8)	76 (50.7)	0.04
Anticholinergic OAB medication use, *n* (%)			0.82
Never	157 (80.1)	116 (77.3)	
Former, no longer using	29 (14.8)	25 (16.7)	
Former, still using	10 (5.15)	9 (6.0)	
Β3-agonist OAB medication use, *n* (%)			< 0.001
Never	167 (85.2)	148 (98.7)	
Former, no longer using	16 (8.2)	2 (1.3)	
Former, still using	14 (6.6)	0 (0)	

Overall adherence to PFPT therapy for the whole study population was low, with 83 (24.0%) completing ≥ 50% of the recommended sessions. Of those 83 patients, 45 (13.0%) reported complete adherence to PFPT therapy. Specifically, patients in the PT group were more likely to complete their PFPT course or attend ≥ 50% of the recommended number of PFPT sessions than patients in the PT + Med group (PT *n* = 60 [30.6%] vs PT + Med: *n* = 23 [15.3%], *p* = 0.001). Additionally, patients in the PT group were more likely to initiate PFPT than the PT + Med group (Table 2). Patients in the PT group attended a higher median number of PFPT sessions (PT 1 session vs PT + Med 0 sessions,* p* < 0.001). There was no difference between groups regarding completion of the entire recommended PT course.

**Table 2:** Adherence to pelvic floor physical therapy (PFPT) and clinical outcomes in patients receiving physical therapy (PT) or physical therapy plus medication (PT + Med) for overactive bladder (OAB)

	PT group, *n* = 196	PT + Med group, *n* = 150	*p* value
Number of PT sessions, median (IQR)	1 (0–6)	0 (0–2.5)	< 0.001
Adherence of ≥ 50% sessions*, *n* (%)	60 (30.6)	23 (15.3)	0.001
Adherence, *n* (%)			< 0.001
Initiation	39 (19.9)	17 (11.3)	0.03
≥ 50%	29 (14.8)	9 (6.0)	0.01
Completion	31 (15.8)	14 (9.3)	0.08
No recorded PFPT sessions	97 (49.5)	110 (73.3)	< 0.001
Any follow-up with urogynecology, *n* (%)	77 (39.3)	81 (55.1)	0.004
Follow-up, months, median (IQR)	7 (4–20)	10.5 (6–18)	0.30
New medication after physical therapy, *n* (%)	26 (28.6)	18 (19.8)	0.17
Any minimally invasive treatment, *n* (%)	8 (4.1)	15 (10.0)	0.03
Onabotulinumtoxin	5 (2.6)	7 (4.7)	0.38
Percutaneous tibial nerve stimulation	1 (0.5)	4 (2.7)	0.17
Sacral neuromodulation	2 (1.0)	4 (2.7)	0.41

The PT + Med group was more likely to follow up with a urogynecologist (PT *n* = 77 [39.3%] vs PT + Med *n* = 81 [55.1%], *p* = 0.004) and progress to minimally invasive therapy (PT *n* = 8 [4.1%] vs PT + Med *n* = 15 [10%], *p* = 0.03). Of the 160 patients who moved onto minimally invasive therapy, 12 received intravesical onabotulinumtoxinA, 5 received percutaneous tibial nerve stimulation, and 6 underwent sacral neuromodulation.

Rates of new medication prescription were similar between those with a previous anticholinergic or beta-3-agonist trial (24.6%, *p* = 0.56), and there was no significant difference in PFPT adherence between those with previous medication use (adherent to ≥ 50% sessions 30.5% vs non-adherence 22.8%, *p* = 0.16).

Using a multivariable logistic regression model to control for BMI, initiation of vaginal estrogen cream, diabetes status, and baseline UDI score, we found that the number of patients completing ≥ 50% of the recommended PFPT sessions remained significantly lower for the PT + Med group (0.38 adjusted odds ratio [aOR], *p* = 0.001, 95% CI 0.21–0.69). Additionally, BMI and UDI scores remained significantly higher for the PT + Med group on the multivariable logistic regression model (Table 3).

**Table 3:** Multivariable logistic regression model of overactive bladder patients treated with medication and pelvic floor physical therapy (PT)

	aOR	*p* value	95% CI
≥ 50% completed PT	0.38	0.001	0.21–0.69
Body mass index	1.05	0.01	1.01–1.09
Estrogen initiation	1.29	0.31	0.79–2.09
Diabetes	1.67	0.16	0.82–3.40
UDI data	1.02	0.009	0.03–0.31

*aOR* adjusted odds ratio, *CI* confidence interval, *UDI* Urogenital Distress Inventory

## Discussion

In this retrospective cohort study of female patients with OAB who were referred for PFPT, the addition of a concomitant prescription for OAB medication was associated with decreased PFPT adherence. Patients with concomitant medication were also more likely to report higher baseline UDI scores, attend no PFPT sessions, and progress to minimally invasive therapy. To our knowledge, this study is the first to evaluate PFPT adherence in patients undergoing PFPT alone versus PFPT with concomitant OAB medication prescription. Our findings establish the groundwork necessary to better understand factors that affect PFPT adherence in OAB patients.

Although PFPT and medication both offer symptom relief, medication tends to provide faster symptom improvement, leading patients to abandon their PFPT regimen. It can take many weeks after initiating PT for patients to perceive a positive impact on their symptoms whereas medications can show improvement in as little as 1 week, with medication-related quality-of-life improvements evident at 4 weeks [7, 17]. Anticholinergics and beta-3 agonists take less time to improve patients’ symptoms, as they are not reliant on neural adaptation [17]. We hypothesized that the initiation of medication might be associated with decreased PFPT adherence, as patients would have symptom improvement from pharmacotherapy before they were able to initiate and/or achieve relief from PFPT, and our findings support that hypothesis.

This is in line with the finding of Venegas et al. that condition-related factors played an important role in long-term adherence to pelvic floor exercise in women with urinary incontinence [18]. Patients who felt that exercises were unnecessary or were no longer suffering from symptoms had poor long-term adherence [18]. We suspect that similarly, patients in our study who were prescribed concomitant OAB medications noted improvement in their symptoms prior to initiating PFPT. Therefore, these patients may have no longer found PFPT necessary or were less motivated to undergo the effort or cost of PFPT given their symptom improvement.

The low rate of PFPT adherence seen in our study is similar to adherence reported in the literature for other disorders. In our study, only 24% of patients completed ≥ 50% of the recommended sessions. This is similar to a study evaluating adherence to PFPT for fecal incontinence, which showed that 64% of patients initiated PFPT but only 20% reported complete adherence [12]. These rates also mirror adherence data for PFPT among patients with high-tone pelvic floor disorders [13]. A retrospective study of PFPT adherence found improved adherence with a multidisciplinary clinic that allows patients to be seen for PFPT the same day as their new urogynecology visit (91% vs 61%); however, this did not improve overall completion rates (49% vs 41%) [14]. More research on the barriers to PFPT adherence and ways to improve patient satisfaction with PFPT are needed.

In evaluating adherence, we looked at how UDI scores correlated with treatment plans and outcomes for the two cohorts. Patients prescribed concomitant OAB medications at the time of PFPT referral had higher UDI scores than patients treated with PFPT alone. Additionally, patients with concomitant medications were more likely to progress to minimally invasive therapies. Higher UDI scores correspond to high symptom burden, and patients with a higher degree of bothersome symptoms are more likely to seek OAB treatment [4]. Therefore, this patient population may be more likely to be prescribed concomitant pharmacotherapy and progress through the treatment algorithm to third-line, minimally invasive therapies. Future studies need to tabulate post-treatment UDI scores so that more accurate comparisons between the PT group and the PT + Med group can be made.

This was a retrospective study and therefore bound by the typical limits inherent in retrospective reviews. We were unable to control for significant differences in patient demographics and clinical characteristics that could influence the primary outcome. Data were limited to what is available in patient medical records, and some records may be incomplete, with missing PFPT records or not including follow-up in different health care system. Our sample of a regionally specific patient population was homogenous and limits generalizability. Owing to the study design, patient preferences and reasons for adherence or non-adherence were not available (i.e., patient motivation, socioeconomic status, barriers to care, etc.). Additionally, we were not able to track medication adherence with this retrospective study design. The wait time between PFPT referral and availability to schedule PT was not available but may influence whether patients initiate PFPT or whether they adhere to it. Future studies are needed to better understand patient preferences regarding OAB treatment and timing of medication initiation.

## Conclusion

Our findings suggest that PT adherence is greater in patients without concomitant OAB medication prescription, but these findings do not include the possible impact of patient treatment preferences or goals. Improving OAB treatment adherence and outcomes is important given the significant personal and societal burdens of OAB. Understanding barriers to PFPT and medication compliance can help to improve patient treatment in the future. Further research is needed to address the factors affecting PFPT attendance and those surrounding concomitant medication usage, with the goal of improving treatment outcomes.

## Data Availability

De-identified data is available for further analysis upon request.

## References

[1] Cameron AP, Chung DE, Dielubanza EJ, Enemchukwu E, Ginsberg DA, Helfand BT, et al. The AUA/SUFU guideline on the diagnosis and treatment of idiopathic overactive bladder. J Urol. 2024;212(1):11–20. 38651651 10.1097/JU.0000000000003985

[2] Reynolds WS, Fowke J, Dmochowski R. The burden of overactive bladder on US public health. Curr Bladder Dysfunct Rep. 2016;11(1):8–13. 27057265 10.1007/s11884-016-0344-9 PMC4821440

[3] Stewart WF, Van Rooyen JB, Cundiff GW, Abrams P, Herzog AR, Corey R, et al. Prevalence and burden of overactive bladder in the United States. World J Urol. 2003;20(6):327–36. 12811491 10.1007/s00345-002-0301-4

[4] Milsom I, Kaplan SA, Coyne KS, Sexton CC, Kopp ZS. Effect of bothersome overactive bladder symptoms on health-related quality of life, anxiety, depression, and treatment seeking in the United States: results from EpiLUTS. Urology. 2012;80(1):90–6. 22748867 10.1016/j.urology.2012.04.004

[5] Lu Z, Zhang J, Lin S, Fan Z, He Z, Tang F. Associations between overactive bladder and sleep patterns: a cross-sectional study based on 2007–2014 NHANES. BMC Urol. 2023;23(1):184. 37957629 10.1186/s12894-023-01329-z PMC10642019

[6] Onukwugha E, Zuckerman IH, McNally D, Coyne KS, Vats V, Mullins CD. The total economic burden of overactive bladder in the United States: a disease-specific approach. Am J Manag Care. 2009;15(4 Suppl):S90–7. 19355803

[7] Wallace SL, Miller LD, Mishra K. Pelvic floor physical therapy in the treatment of pelvic floor dysfunction in women. Curr Opin Obstet Gynecol. 2019;31(6):485–93. 31609735 10.1097/GCO.0000000000000584

[8] Bykoviene L, Kubilius R, Aniuliene R, Bartuseviciene E, Bartusevicius A. Pelvic floor muscle training with or without tibial nerve stimulation and lifestyle changes have comparable effects on the overactive bladder. A randomized clinical trial. Urol J. 2018;15(4):186–92. 29781068 10.22037/uj.v0i0.4169

[9] Adams SR, Dessie SG, Dodge LE, Mckinney JL, Hacker MR, Elkadry EA. Pelvic floor physical therapy as primary treatment of pelvic floor disorders with urinary urgency and frequency-predominant symptoms. Female Pelvic Med Reconstr Surg. 2015;21(5):252–6. 26313494 10.1097/SPV.0000000000000195

[10] Monteiro S, Riccetto C, Araújo A, Galo L, Brito N, Botelho S. Efficacy of pelvic floor muscle training in women with overactive bladder syndrome: a systematic review. Int Urogynecol J. 2018;29(11):1565–73. 29644384 10.1007/s00192-018-3602-x

[11] Shannon MB, Genereux M, Brincat C, Adams W, Brubaker L, Mueller ER, Fitzgerald CM. Attendance at prescribed pelvic floor physical therapy in a diverse urban urogynecology population. PM R. 2018;10(6):601–6. 29138041 10.1016/j.pmrj.2017.11.008

[12] Ross JH, Sinha A, Propst K, Ferrando CA. Adherence to pelvic floor physical therapy referrals in women with fecal incontinence. Female Pelvic Med Reconstr Surg. 2022;28(3):e29–33. 35272329 10.1097/SPV.0000000000001140

[13] Woodburn KL, Tran MC, Casas-Puig V, Ninivaggio CS, Ferrando CA. Compliance with pelvic floor physical therapy in patients diagnosed with high-tone pelvic floor disorders. Female Pelvic Med Reconstr Surg. 2021;27(2):94–7. 31045618 10.1097/SPV.0000000000000732

[14] Brown HW, Barnes HC, Lim A, Giles DL, McAchran SE. Better together: multidisciplinary approach improves adherence to pelvic floor physical therapy. Int Urogynecol J. 2020;31(5):887–93. 31463525 10.1007/s00192-019-04090-w PMC7047562

[15] Kasman A, Stave C, Elliott CS. Combination therapy in overactive bladder-untapped research opportunities: a systematic review of the literature. Neurourol Urodyn. 2019;38(8):2083–92. 31483070 10.1002/nau.24158

[16] Mattiasson A, Masala A, Morton R, Bolodeoku J; SOLAR Study Group. Efficacy of simplified bladder training in patients with overactive bladder receiving a solifenacin flexible-dose regimen: results from a randomized study. BJU Int. 2010;105(8):1126–35. 19818077 10.1111/j.1464-410X.2009.08910.x

[17] Chapple CR, Nitti VW, Khullar V, Wyndaele JJ, Herschorn S, van Kerrebroeck P, et al. Onset of action of the β3-adrenoceptor agonist, mirabegron, in Phase II and III clinical trials in patients with overactive bladder. World J Urol. 2014;32(6):1565–72. 24458878 10.1007/s00345-014-1244-2 PMC4236626

[18] Venegas M, Carrasco B, Casas-Cordero R. Factors influencing long-term adherence to pelvic floor exercises in women with urinary incontinence. Neurourol Urodyn. 2018;37(3):1120–7. 29095511 10.1002/nau.23432

